# Dr. Oliver's Journal

**Published:** 1881-11

**Authors:** 


*Dr. Oliver's Journal* replies to a notice made of it some time ago by the writer. We confess to have made a mistake, one which led us into error. It may be illustrated by the story of the old methodist preacher who said, that on one occasion the services were interrupted and the congregation annoyed by the unseemingly behavior of a person in the audience, to which person the preacher delivered a severe rebuke. He learned subsequently to his sorrow that the disorderly young man was troubled with congenital *mollities cerebri*—was naturally "soft." We apologise—to our readers. H.

